# The relationship between negative peer relationship and non-suicidal self-injury in Chinese adolescents: A moderated-mediation model

**DOI:** 10.3389/fpsyg.2022.913872

**Published:** 2022-08-17

**Authors:** Jia-bin Xu, Na Jiang, Qin Qin, Qin Jiang

**Affiliations:** The School of Health, Fujian Medical University, Fuzhou, China

**Keywords:** non-suicidal self-injury, negative peer relationship, regulatory emotional self-efficacy, adolescents, gender

## Abstract

**Objective:**

The objective of the study was to investigate the mediating effect of regulatory emotional self-efficacy (RESE) between negative peer relationship and non-suicidal self-injury (NSSI), as well as the moderating effect of gender difference.

**Methods:**

A study of 578 Chinese adolescents (46.9% males, mean age = 16.32 years, SD =0.54) was conducted using the Child and Adolescent Peer Relationship Inventory, Regulatory Emotional Self-Efficacy Scale, and the Adolescent Self-Injury Questionnaire.

**Results:**

Negative peer relationship was positively correlated with NSSI, and RESE was negatively correlated with negative peer relationship and NSSI. RESE mediated the association between negative peer relationship and NSSI. The first stage(predicting the effect of negative peer relationship on RESE) and the second stage (predicting the effect of RESE on NSSI) of the mediation effect of RESE were both moderated by gender. Specifically, compared with boys, the effect of negative peer relationship on RESE was stronger for girls, and the effect of RESE on NSSI was stronger for girls than boys.

**Conclusion:**

Negative peer relationships may influence NSSI of adolescents through the mediating effect of RESE and the moderating role of gender.

## Introduction

Non-suicidal self-injury (NSSI) was a behavior where individuals deliberately used any means possible to hurt themselves. This behavior often was performed without the intent of committing suicide and was not considered socially acceptable (Klonsky and Olino, 2008). NSSI was relatively common in adolescents (Nock, 2010). The incidence of NSSI among Chinese adolescents was generally higher than adolescents in western countries. Two meta-analyses showed that the incidence of NSSI among adolescents in China was 22.37%–27.4% (Han et al., 2017; Lang and Yao, 2018). NSSI was associated with many mental health issues including depression and anxiety (Bentley et al., 2015). Researchers had confirmed that the physical development, such as the prefrontal brain of middle school students at this stage, was becoming mature, and the brain was especially susceptible to environmental influences (Herd and Kim-Spoon, 2021). Adolescents at this stage had poor psychological state and were impulsive, unable to correctly deal with their negative emotions, and were prone to some extreme NSSI behaviors (Yu et al., 2013). Therefore, it was important to explore the factors’ influencing NSSI in adolescents.

Peer relationship referred to an interpersonal relationship established and developed in the process of communication between peers at the same level of psychological development, which contained the meaning, expectations, and emotions generated in a series of interactions (Rubin et al., 1998). Peer relationship was of great importance to children’s social development and peer relationship affecting many aspects of their mental process and behaviors. High-quality peer relationships contributed to the healthy development of teenagers’ learning, cognition, emotion, and personality. High-quality peer relationship was correlated with adolescents’ positive emotions (Lan and Wang, 2019). A good and harmonious peer relationship could promote the level of individual self-concept (Maunder and Monks, 2019). However, negative peer relationship might lead to adjustment difficulties, emotional disorders, and even high-risk behaviors (Zhang, 1999). Students with negative peer relationships were less developed in emotional expression than those with high-quality peer relationships (Sun et al., 2007). The development of adolescent antisocial behavior was influenced by negative peer relationship, and peer rejection was significantly related to aggression (Kim and Nho, 2007; Ji et al., 2017). Moreover, negative peer relationship was considered an important factor of NSSI in many theoretical models. According to the interpersonal/system model, the dysfunctional interpersonal environment of an individual was considered to be the main factor leading to NSSI (Messer Julia and Fremouw, 2008). Based on the four-function model of NSSI, NSSI could be maintained by interpersonal positive reinforcement, in which the behavior was followed by the occurrence or increase in a desired social event, such as support from others. It could be maintained by interpersonal negative reinforcement, in which the behavior was followed by a decrease or cessation of some social events, for example, peers stop bullying (Nock, 2010). Many research supported interpersonal/system model and four-function model of NSSI. The research showed that peer victimization (referring to the repeated rejection, attack, and humiliation of individuals from the group) was an important predictor of NSSI (Geel et al., 2015; Madden et al., 2018). School bullying and peer rejection increased the possibility of NSSI (Esposito et al., 2019). A longitudinal study also showed that negative parent–child relationship and negative peer relationship were risk factors for future NSSI in adolescents (Victor et al., 2019). In addition, two studies had shown that low-quality peer relationship was a risk factor for NSSI among adolescents (Yurkowski et al., 2015; Li et al., 2021). Previous studies had explored the impact of interpersonal risk factors such as peer victimization, bullying, peer exclusion, and cyberbullying on NSSI. Few studies had focused on the adolescents who had low peer support and lacked interpersonal communication and friendship. Although this condition was not as serious as peer victimization or bullying, this long-term and continuous situation could not be ignored. It might lead to loneliness, inferiority, depression, and other emotions of teenagers (Yang, 2018). This study explored the impact of negative peer relationships on NSSI, which was not serious but common among adolescents. Based on the NSSI integrated theoretical model (Nock, 2010), the main function of NSSI was emotion regulation, which could separate individuals from negative and painful emotions and maintain NSSI through continuous negative reinforcement. Many studies had shown that non-adaptive and negative cognitive emotion regulation strategies were significantly positively correlated with NSSI (Haid-Stecher and Sevecke, 2019; Brereton and McGlinchey, 2020). People who engaged in NSSI often reported greater emotion dysregulation than those without any NSSI history (Andover and Morris, 2014). Although researchers agreed that people who engaged in NSSI had emotion dysregulation, few studies had explored the causes of their emotion dysregulation. Regulatory emotional self-efficacy (RESE) might be an answer. RESE referred to an individual’s confidence level and perceived ability in regulating emotions (i.e., positive and negative emotions; Caprara et al., 1999). It was considered the core of emotion regulation. Individuals with stronger RESE had better emotion regulation ability and emotional state (Bandura et al., 2003). Weaker RESE might also affect individual mental health, affect the improvement of subjective wellbeing and prosocial behaviors, and might lead to depression, criminal behavior, addiction, and other effects (Tang et al., 2010). An empirical research had supported the protective role of RESE against NSSI (Liu et al., 2020).

Based on Caprara and Steca (2005), regulatory emotional self-efficacy was closely related to interpersonal relationships. People with better quality of interpersonal relationships were more confident about effectively dealing with emotion and interpersonal communication. In addition, according to self-efficacy theory of Bandura (1999), individuals’ perception of their own abilities was affected by the evaluation of people around them. Especially the opinions of important others, which could affect individuals’ self-efficacy, and then affect individuals’ negative emotional responses and non-adaptive behaviors. These results suggested that RESE, as an important variable of self-efficacy, might mediate the relationship between interpersonal relationship (such as peer relationship) and individuals’ NSSI. Gender was an important factor affecting all aspects of adolescents’ physical and mental health. Many researchers had indicated differences between male and female groups in RESE and NSSI. Researchers confirmed that the incidence rate of NSSI among females was higher than that in males (Nock, 2010; Bresin and Schoenleber, 2015; Gandhi et al., 2015; Wan et al., 2020). It indicated that females might be more likely than males to resort to NSSI to relieve overwhelming, painful emotions. In addition, the research found that there was a significant gender difference in emotional regulation self-efficacy in adolescence (Bandura et al., 2003). On one hand, compared with female, male could deal with negative emotions with more self-efficacy of emotion regulation in adolescent, but after entering into late adulthood, male shows less self-efficacy of emotion regulation than female. On the other hand, female’s self-efficacy in dealing with negative emotions gradually increased from early adulthood to old age, while both female and male’s self-efficacy in expressing positive emotion decreased as they grew old (Tang et al., 2010). Adolescent girls were more sensitive to emotions than boys, meaning they were more likely than boys to perceive and absorb negative emotions (Garaigordobil, 2020). This reduced their self-efficacy in dealing with negative emotions. The weakness in emotional regulation self-efficacy was likely to cause females to be less able to relieve the negative emotions caused by negative peer relationship, then they regulated painful emotions through NSSI. Based on this, this study considered the impact of gender in RESE and NSSI.

**Figure 1:**
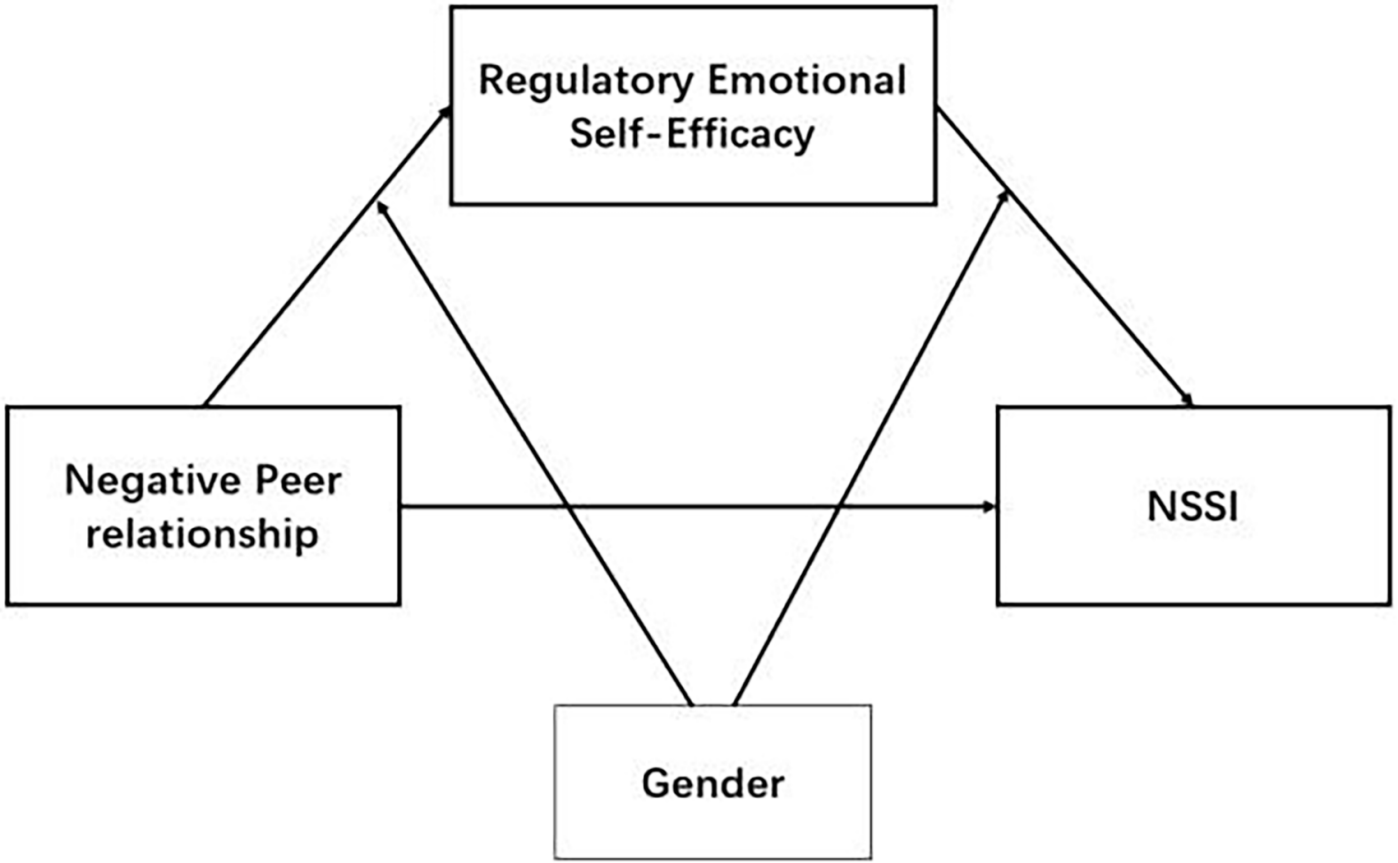
The proposed moderated-mediation model of the relationship between peer relationship and non-suicidal self-injury (NSSI).

The purpose of the present study was twofold. First, this study examined whether RESE mediated the association between negative peer relationship and NSSI in a sample of Chinese adolescents. Based on self-efficacy theory, we proposed the hypothesis that RESE mediates the association between negative peer relationship and adolescents’ NSSI. Second, the study explored the moderating role of gender. Given existing research, we hypothesized that gender played a moderating role in the relationship between negative peer relationship and RESE, and played a moderating role between RESE and NSSI. Specifically, compared with males, females’ RESE would be more influenced by negative peer relationship, and compared with male, female’s NSSI would be more influenced by RESE. To sum up, on the basis of interpersonal/system model and self-efficacy theory, the current study explored a mediation model between peer relation, RESE, and NSSI, and examined the moderating effect of gender. This study would contribute to an enhanced understanding the mechanism of negative peer relationship on adolescent NSSI and provide insight regarding the prevention strategies and methods of intervention. Figure 1 illustrates the proposed moderated-mediation model.

## Materials and methods

### Participants

The participants were 612 high school students, who were selected using a convenient sampling method from a public middle school in Fuzhou City, Fujian Province of China. Fuzhou City was the capital of Fujian Province, which was located in the southeast coast of China. The questionnaires were distributed to the students in class. Five hundred and seventy eight valid questionnaires were obtained, and the effective recovery rate was 94.44%. Among them, there were 307 male students (53.10%) and 271 female students (46.9%). Three hundred and seventy seven of them were 10th grade students (62.20%) and 201 of them were 11th grade students (34.80%). All subjects were given informed consent.

### Measures

#### Negative peer relationship

Child and Adolescent Peer Relationship Inventory was used in this study. This scale was developed by Guo and Zhang (2004) to measure individuals’ perception of their peer relationship. The scale contains a total of 22 items, including seven reverse scoring items (e.g., “I feel unhappy in school,” “I do not like many things about school,” etc.) and 15 positive scoring items (e.g., “I pay attention to how other students view me,” “my classmates treat me well,” etc.). Each item was rated on a four-point scale, ranging from “1 = not like this” to “4 = always like this.” The higher the total score of the scale was, the worse the peer relationship was. Cronbach’s alpha in this study was 0.75.

#### Regulatory emotional self-efficacy

The 17-item Chinese version of the scale was revised by Wang et al. (2013) based on an existing regulatory emotional self-efficacy scale (RESES; Caprara et al., 2008) that includes two subscales: Perceived self-efficacy in expressing positive effect (POS, e.g., “express joy when good things happen to you”) and perceived self-efficacy in managing negative effect (NEG, e.g., “keep away from getting discouraged by strong criticism”). Responses were made on a five-point scale, ranging from 1 = not well at all to 5 = very well. The higher the total score of the scale was, the higher level of self-efficacy of emotion regulation was. In this study, the Cronbach’s alpha of total scale was 0.896. POS sub-scale is 0.853 and NEG sub-scales is 0.906.

#### Non-suicidal self-injury

The adolescent self-injury questionnaire was revised by Yu (2013) on the basis of the existing scale. The questionnaire consisted of one open question and 18 items, measuring two parts of NSSI—the frequency of NSSI (e.g., In the past year, have you engaged in the following behaviors to deliberately harm yourself without suicidal intent?) and degrees of physical harming behavior (e.g., how serious is the physical harm caused by this behavior?). The frequency items were rated on four levels: never, once, twice to four times, and above five times, which were scored from “0” to “3,” respectively. The degrees of physical harming items were rated on five levels: none, mild, moderate, severe, and extremely severe. The total score was the product of the score for frequency and the score for degrees. The higher the total score was, the more severe NSSI was. In this study, whether the self-injury level was “0” or not was taken as the criterion for judging whether there was NSSI or not. In this study, Cronbach’s α was 0.877.

### Procedure

The study was implemented in compliance with the principles of the Declaration of Helsinki. The Biomedical Research Ethics Committee of Fujian Medical University approved this study content and data collection procedures(No. 89). The data were collected in the classrooms of a public middle school between October and November of 2019. Informed consent was obtained from school administrators, students, and their parents before the data collection. Questionnaires were distributed in class. It took about 15 min to complete all the questionnaires. Students were informed about the confidentiality of the collected data, and their right to quit at any time if they did not wish to answer the questionnaires. Furthermore, all participants were taught with the knowledge about emotion management and were provided with psychological hotline service for free in the following 1 month.

### Statistical analyses

In this study, SPSS26.0 software was used for the statistical analysis of the data. First, the relationships between variables were calculated through correlation analysis, and the correlation between gender and other variables was calculated by point biserial correlation. Second, model 4 in SPSS macro-process version 3.0 compiled by Hayes (2013) was used to test the mediating effect of RESE between negative peer relationship and NSSI. Finally, Model 58 in SPSS macro-process version 3.0 compiled by Hayes (2013) was used to analyze the moderating mediation model. All the continuous variables were standardized, and the interaction terms were computed from these standardized scores. The bootstrapping method produced 95% bias-corrected confidence intervals of these effects from 5,000 resamples of the data. Confidence intervals that did not contain zero indicated significant effects.

## Results

### Analysis on the detection rate and gender difference of adolescent NSSI

Frequency analysis showed that 201 of 578 adolescents had NSSI, with a detection rate of 34.8%. Among them, 95 were male students and 102 were female students. The severity level of self-injury of girls was significantly higher than that of boys (*t* = −2.24, *p* < 0.05), and the level of regulatory emotional self-efficacy of girls was significantly lower than that of boys (*t* = 3.01, *p* < 0.05).

### Descriptive and correlation analyses

Pearson correlation analysis showed that there was a significant positive correlation between negative peer relationship and NSSI; regulatory emotional self-efficacy was significantly negatively associated with negative peer relationship and NSSI, as shown in Table 1. The results suggested that there was a close relationship between negative peer relationship, regulatory emotional self-efficacy, and NSSI.

**Table 1:** Correlation analysis between variables.

	M	SD	1	2	3	4
1. Negative peer relationship	38.75	7.40	1			
2. RESE	62.34	10.27	−0.27^**^	1		
3. NSSI	2.55	6.69	0.26^**^	−0.40^**^	1	
4. Gender	1.47	0.50	−0.160	0.50^**^	−0.45^*^	1

RESE, Regulatory emotional self-efficacy; NSSI, Non-suicidal self-injury.

* *p* < 0.05 and

** *p* < 0.01.

### Negative peer relationship and NSSI: Testing for moderated-mediation

We adopted models 4 and 59 in SPSS macro-process version 3.0 compiled by Hayes to examine the mediating effect of regulatory emotional self-efficacy between negative peer relationship and adolescent self-injury and the moderating effect of gender. In this test, the bias-corrected bootstrap method was used to estimate confidence intervals for mediating and moderating effects by drawing 5,000 samples. The results of the mediating test showed that negative peer relationship had a significant negative predictive effect on regulatory emotional self-efficacy (β = −0.37, *p* < 0.001), and a significant positive predictive effect on NSSI (β = 0.15, *p* < 0.001, *p* < 0.001). Further, regulatory emotional self-efficacy had a significant negative predictive effect on NSSI (β = −0.23, *p* < 0.001). This suggested that regulatory emotional self-efficacy played a partial mediating role in the influence of negative peer relationship and NSSI, accounting for 37.5% of the total effect. The results were shown in Table 2.

**Table 2:** The mediating role of RESE.

Mediating variable	Effect	Effect size	Effect ratio	Boot standard error	95% CI
RESE	total effect	0.26^**^		0.04	(0.18, 0.34)
	direct effect	0.17^**^		0.04	(0.09, 0.26)
	indirect effect	0.09^**^	34.6%	0.03	(0.05, 0.15)

RESE, Regulatory emotional self-efficacy.

** *p* < 0.01.

In addition, after adding gender as a moderating variable in the mediation model, the results showed that the interaction of negative peer relationship × gender had a significant negative predictive effect on regulatory emotional self-efficacy (β = −0.09, *p* < 0.05), and that was, gender negatively moderated the relationship between negative peer relationship and regulatory emotional self-efficacy. In addition, the interaction of regulatory emotional self-efficacy × gender had a significant negative predictive effect on NSSI (β = −0.12, *p* < 0.05), and that was, gender negatively moderated the relationship between regulatory emotional self-efficacy and NSSI, the results are shown in Table 3.

**Table 3:** The moderated-mediating effect of negative peer relationship on NSSI.

Outcome variable	predictor variable	*R*	*R* ^2^	*F*	*β*	95% CI	*t*
RESE	negative peer relationship	0.30	0.09	19.57^**^	−0.26	(−0.34, −0.18)	−6.53^**^
	gender				−0.11	(−0.19, −0.04)	−2.85^**^
	negative peer relationship*gender				−0.09	(−0.17, −0.01)	−2.26^*^
NSSI	negative peer relationship	0.45	0.20	28.76^**^	0.15	(0.08, 0.23)	3.96^**^
	RESE				−0.35	(−0.43, −0.28)	−8.98^**^
	gender				0.04	(−0.03, 0.12)	1.12
	negative peer relationship*gender				0.01	(−0.07, 0.09)	0.27
	RESE*gender				−0.12	(−0.19, −0.04)	−2.93^*^

NSSI, Non-suicidal self-Injury; RESE, Regulatory emotional self-efficacy.

* *p* < 0.05 and

** *p* < 0.01.

**Figure 2:**
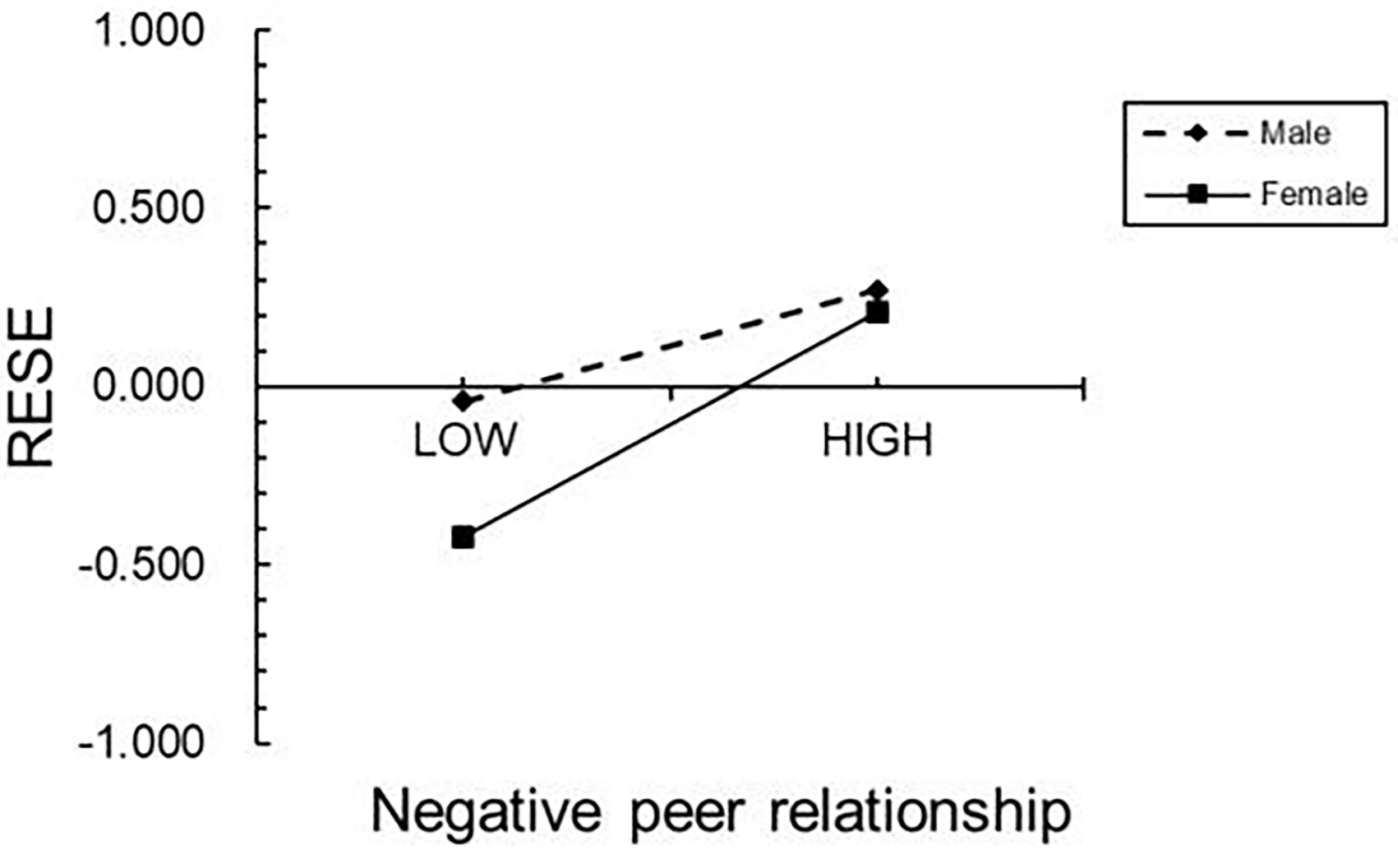
Model of the test for simple slopes showing the moderating influence of gender of the association between negative peer relationship and RESE.

**Figure 3:**
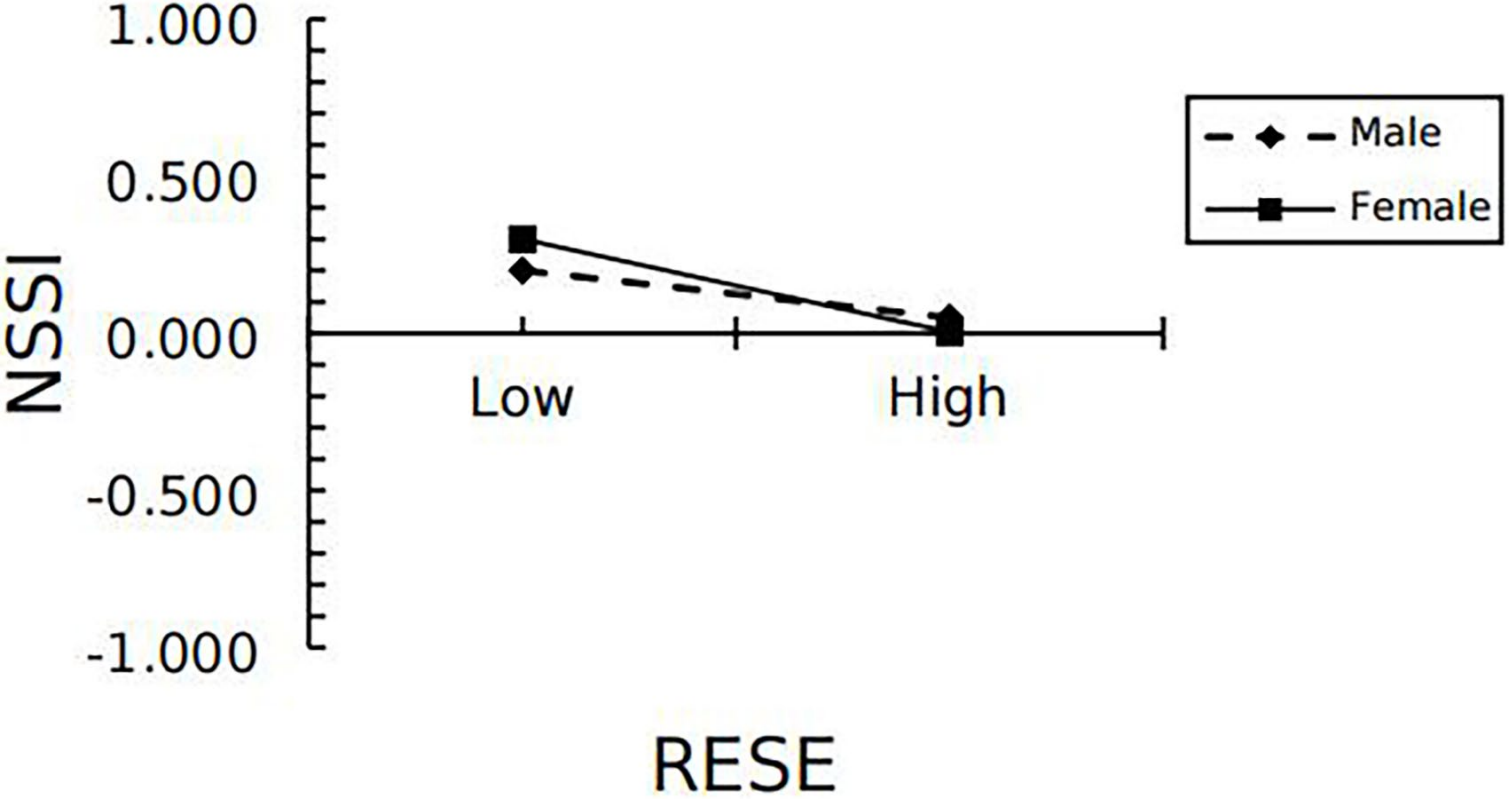
Model of the test for simple slopes showing the moderating influence of gender of the association between RESE and NSSI.

The final mediation moderation model determined that gender moderated the first half of the pathway (from negative peer relationship to regulatory emotional self-efficacy) and the second half of the pathway (from regulatory emotional self-efficacy to NSSI) of the mediating effect of regulatory emotional self-efficacy, verifying the hypothesis of this study. Further, a simple slope test showed that compared with boys (βsimple = 0.24, *p* < 0.001), negative peer relationship had a more significant impact on girls’ regulatory emotional self-efficacy (βsimple = 0.48, *p* < 0.01; see Figure 2). In addition, the effect of regulatory emotional self-efficacy on girls’ self-injury (βsimple = 0.36, *p* < 0.001) was also more significant than that on boys’ self-injury (βsimple = 0.18, *p* < 0.001; see Figure 3).

## Discussion

This study investigated the relationship between negative peer relationship and NSSI. We examined a moderated mediation model of NSSI with one risk factor of NSSI (negative peer relationship) and one protective factor of NSSI (RESE) in a large sample of Chinese adolescents. The results showed a significant positive correlation between negative peer relationships and NSSI. It meant that the worse the quality of peer relationship was, the higher the probability and frequency of NSSI in adolescents was. This was consistent with previous studies (Concetta et al., 2019; Wu et al., 2021). Negative peer relationships led to poor interpersonal relationships with classmates, estrangement from peers, and lack of companionship from classmates as seen in the study and in life. This result was consistent with the theory of interpersonal relationship/system model meaning that the risk of NSSI was higher in individuals who were in a relatively dysfunctional external environment where it was difficult to meet the needs of individual communication and belonging (Messer Julia and Fremouw, 2008). In addition to the external environment, young people were experiencing the rapid development of the nervous system and brain regions associated with emotional processing, such as the cingulate cortex and the amygdala. However, the development of their cognitive control system, such as the prefrontal cortex was relatively lagging, meaning that teens’ cognitive thinking and ability to regulate emotion was weaker (Elizabeth et al., 2016). As a result, adolescents might have more difficulty dealing with negative emotions such as depression, anxiety, anger, and loneliness caused by peer relationships. Individuals with negative peer relationships also had fewer opportunities to alleviate negative emotions through self-disclosure or peer support, which further increased the impact of negative emotions.

### The mediating role of regulatory emotional self-efficacy

This study confirmed our hypothesis. The result showed that RESE partly mediated the association between negative peer relationship and adolescents’ NSSI. Negative peer relationship could affect NSSI through RESE. In other words, negative peer relationship would reduce the level of RESE, and the decrease of RESE might further increase the possibility or frequency of NSSI. Thus, a decrease of RESE might serve as one explanatory mechanism for the relationship between negative peer relationship and NSSI in teenagers. This result was also consistent with the emotion regulation theory of NSSI. RESE reflected an individual’s confidence in managing and expressing emotions. It also was considered as core factor of emotion regulation ability, which was closely related to the reduction and control of individual negative emotions (Mesurado et al., 2018). Zhang et al. (2019) found that low RESE could lead to depression, negative coping style, low subjective wellbeing, and other adverse psychological behavior consequences. When an individual could not support the function of regulating negative emotions, the possibility of using external negative coping methods (i.e., substance abuse, alcohol dependence, NSSI, etc.) would be greatly enhanced (Siodmok et al., 2013; Azizi et al., 2019; Gratz Kim et al., 2020). The influence of external factors such as negative peer relationship and internal factors such as immature of emotional regulation ability indicated that those adolescents were more likely to use NSSI to relieve accumulated and unbearable negative emotions. In addition to the mediation results, each of the individual pathways in this mediation model was noteworthy. First, negative peer relationship was found to have a significant direct effect on both RESE and NSSI. This confirmed the negative effects of low-quality peer relationship in Chinese adolescents and provided empirical support regarding the effect of negative peer relationship on the risk of NSSI. For social functioning of adolescents, peer relationships were even greater important than family relationships (Badenes-Ribera et al., 2019). In negative peer relationships, adolescents were more likely to produce negative emotions. These might internalize into an individual negative cognition, such as self-criticism in those adolescents unable to properly deal with their negative emotions (Marian et al., 2021). In the end, they regulated their emotions through NSSI. Second, RESE was a significant negative predictor for adolescents’ NSSI, which was in accordance with existing literature (Gratz Kim et al., 2020; Guo et al., 2021; Liu et al., 2021). In this model, RESE was the strongest predictor for NSSI. Based on the self-efficacy theory, self-efficacy was the core of emotion regulation ability, and individuals who had adaptive emotion regulation could mitigate the effects of negative emotions without any need for NSSI (Li and Li, 2022).

### The moderating role of gender

The present study confirmed the moderating role of gender, not only in the indirect association between negative peer relationship and RESE, but also in the association between RESE and NSSI. The difference test found that adolescent females had lower RESE and higher NSSI than those males. The simple slope test indicated that female adolescents’ RESE was more affected by negative peer relationship compared to males. Specifically, female adolescents with negative peer relationships had lower level of RESE than males with negative peer relationships. Based on social role theory, females paid more attention to interpersonal relationships than males (Eagly and Wood, 2012), and were vulnerable to peer relationship. The research reported that female adolescents tended to perceive greater daily peer hassles than male adolescents (Xavier et al., 2018). When female adolescents were unable to form close, supportive relationship with their peers, they were more likely to suffer from loneliness and depression. Female adolescents also had higher emotion reactivity and experienced more negative emotions and rumination than males in adolescence (Bandura et al., 2003; Johnson and Whisman, 2013). Rumination, avoidance, dissociation, and depressive symptoms in female adolescents were greater than those in male adolescents when experiencing daily distress like peer arguments (Greco et al., 2008; Howe-Martin et al., 2012; Biglan et al., 2015; Xavier et al., 2018).All of these reduced their confidence to regulate their emotions. Thus female adolescents had lower RESE than males when experiencing negative peer relationship.

Compared with male adolescents, the predictive effect of RESE on NSSI was more significant in female adolescents. There might be many reasons for this finding. Many research showed that the susceptibility of females to NSSI was significantly higher than that of males (Garaigordobil, 2020; Jeong and Kim, 2021; Li et al., 2021). At the same level of interpersonal risk factors, female would be more prone to NSSI. Moreover, research showed that males had higher self-efficacy in regulating negative emotions (Tang et al., 2010; He et al., 2019). The reason might be that males preferred cognitive reappraisal strategies, while females preferred emotion-focused strategies, which easily led to stronger negative emotional susceptibility of females(McRae et al., 2008). Studies from neuroimaging also supported gender differences in emotion regulation. Mak et al. (2009) used fMRI to explore gender differences in the process of emotion regulation and found that females were better at regulating positive emotions than males, while males were better at regulating negative emotions than females. This indicated that males were more likely to control and alleviate the effects of negative emotions and then reduced the occurrence of NSSI. Although some studies found females had higher positive emotion regulation ability than males (He et al., 2019; Xu, 2021), the emotional regulation function of NSSI emphasized its role in managing negative emotions for the purpose of emotional control (Messer Julia and Fremouw, 2008). While males were more likely to engage in emotional regulation strategies in response to negative peer relationship, adolescent females were more likely to engage in NSSI to deal with internal distress emotion.

### Limitations and practical implications

There were several limitations to this study. First, due to the cross-sectional design of this study, we were unable to make any causal inferences about the observed associations. Future research should use longitudinal studies to better define the paths in our theoretical model. Second, the results of this study were based on self-reported results, and individuals might hide their self-injury behaviors. Moreover, social desirability, respondent bias, and recall bias might influence the results. Future work should use multiple-method assessment. Third, only 10th grade students and 11th grade students in one middle school were selected, while students in others grades or in other schools were not involved. So, the caution was warranted in generalizing the study findings. Further studies should examine the model in different grades and schools. Finally, RESE was a complex structure consisting of several components (Caprara et al., 2008). The effects of components of RESE on NSSI had not been examined in this study. Future research could focus on the relationship between each specific component of RESE and NSSI and find the strongest protective factors for NSSI to facilitate the development of appropriate interventions.

This was the first study to investigate the relationship between negative peer relationship and NSSI among Chinese adolescents. This study identified a significant moderated-mediation model that explained the effect of negative peer relationship on adolescents’ NSSI, which integrated the Interpersonal/System Model and the Self-Efficacy Theory. This model provided information about the relationship between negative peer relationship and adolescents’ NSSI and offered corresponding empirical evidence for theories about the emotion regulation of NSSI. It also offered some potential methods for preventing the deleterious consequences of negative peer relationship for adolescents. According to these results, regulatory emotional self-efficacy was a vital mediating variable in the relationship between negative peer relationship and adolescents’ NSSI. It provided a new path for the influence of negative peer relationship on NSSI. Further, this model emphasized that gender played an important role in adolescents’ mental health and females were more likely to be influenced by negative peer relationship and RESE than males. Future studies should continue to consider the role of gender in NSSI.

The results of this study had important practical implications. First, the results of this study emphasized the harmful impact of negative peer relationship on adolescents. Intervention programs should be used as much as possible to promote peer understanding and communication. Second, based on the Interpersonal/System Model and the Self-Efficacy Theory, we verified the mediating role of RESE between negative peer relationship and NSSI. The results suggested that by increasing adolescents’ emotion regulation self-efficacy, they could learn to cope effectively with the emotional distress caused by negative peer relationship experiences. Third, we found that gender had a significant difference in the impact of negative peer relationship on NSSI. Thus, teachers and parents should pay attention to adolescent girls, especially in bad peer relationships. At the same time, researchers should also explore and apply the cultivation and improvement of RESE. Dialectical behavior therapy (DBT) had been widely used in NSSI and had shown beneficial effects. More importantly, DBT might be beneficial for adolescents with a wide range of emotional regulation difficulties (MacPherson et al., 2013). Schools could consider psychological courses to teach adolescents to learn emotional regulation skills from DBT.

## Conclusion

This study constructed a moderated mediation model to test the mediating role of RESE in negative peer relationship and NSSI relationship, as well as the moderating role of gender in this mediating role. The first stage (predicting the effect of negative peer relationship on RESE) and the second stage (predicting the effect of RESE on NSSI) of the mediation effect of RESE were both moderated by gender. Compared with boys, the effect of negative peer relationship on RESE was stronger for girls, and the effect of RESE on NSSI was stronger for girls than boys. As negative peer relationship had a negative impact on adolescents, it was necessary to pay attention to those in the negative peer relationships. Interventions should be taken to improve RESE of adolescents. Special attention should be given to adolescent girls.

## Data availability statement

The raw data supporting the conclusions of this article will be made available by the authors, without undue reservation.

## Ethics statement

The studies involving human participants were reviewed and approved by the Biomedical Research Ethics Committee of Fujian Medical University. Written informed consent to participate in this study was provided by the participants' legal guardian/next of kin.

## Author contributions

QJ designed the theoretical framework. J-bX analyzed the data. NJ collected the data. All authors contributed to the article and approved the submitted version.

## Funding

This study was supported by the Social Science Planning Project of Fujian Province (FJ2019B173), Natural Science Foundation of Fujian Province (2021 J01816), and School of Health of Fujian Medical University.

## Conflict of interest

The authors declare that the research was conducted in the absence of any commercial or financial relationships that could be construed as a potential conflict of interest.

## Publisher’s note

All claims expressed in this article are solely those of the authors and do not necessarily represent those of their affiliated organizations, or those of the publisher, the editors and the reviewers. Any product that may be evaluated in this article, or claim that may be made by its manufacturer, is not guaranteed or endorsed by the publisher.
